# ASO Author Reflections: Predicting Early Recurrence After Trimodality Therapy for Esophageal Adenocarcinoma

**DOI:** 10.1245/s10434-018-7016-2

**Published:** 2018-11-15

**Authors:** Lucas Goense, Steven H. Lin

**Affiliations:** 1 Department of Surgery, University Medical Center Utrecht, Utrecht, The Netherlands; 2 Department of Radiation Oncology, The University of Texas MD Anderson Cancer Center, Houston, TX USA

## Past

Surgical resection of the esophagus combined with neoadjuvant chemoradiotherapy (trimodality therapy) is a generally recommended treatment strategy with curative intent for patients with non-metastasized esophageal cancer.^1^ However, even after trimodality therapy, 5-year overall survival remains relatively poor (36–47%), which is mainly attributable to the high incidence (49–85%) of disease recurrence after surgery.^1^ As such, in the group of patients who experience early disease recurrence within 1 year of completing their treatment, the benefit of surgery probably does not outweigh its potential side effects.^2^ Some suggest that consideration should therefore be given to forgo surgery in patients who are likely to have early disease recurrence after surgery. To guide treatment decision making, this study aimed to develop a preoperative prediction model to identify patients at high risk of early disease recurrence after trimodality therapy for esophageal adenocarcinoma.

## Present

The current study identified gender, poor tumor differentiation grade, signet ring cell adenocarcinoma, baseline clinical nodal status, and SUV_max_ of the initial positron emission tomography/computed tomography as independent prognostic factors for early recurrence after trimodality therapy.^3^ Based on the subsequent prediction model, patients at high-risk of early recurrence had no survival benefit of trimodality therapy compared with definitive chemoradiotherapy. This finding suggests that, indeed, a group of patients with clinically operable disease may not benefit from an esophageal resection after chemoradiotherapy.

## Future

An important caveat of this prediction model based on clinicopathological characteristics is that it is not 100% accurate. As such, avoiding surgery based on its predictions may cause the very undesirably result of a missed opportunity for cure in patients with locoregional disease only. Further improvement in prognostication is desired to accurately stratify patients by potential benefit of surgery balanced with risk of occult distant metastases. In this context, quantification of circulating tumor DNA represents a novel approach for disease burden quantification that has the potential to complement prognostication in esophageal cancer. Furthermore, recent studies have shown that functional magnetic resonance imaging^4^ and cancer stem-cell molecules^5^ hold considerable promise for improving the accuracy of prognosis. Future improvement of the current model with these novel predictors will likely improve prognostication, and therewith support choices of care and shared decision making in the treatment of patients with esophageal cancer.
